# Patterns of Carpel Structure, Development, and Evolution in Monocots

**DOI:** 10.3390/plants12244138

**Published:** 2023-12-12

**Authors:** Margarita V. Remizowa, Dmitry D. Sokoloff

**Affiliations:** Biological Faculty, M.V. Lomonosov Moscow State University, 119234 Moscow, Russia; sokoloff-v@yandex.ru

**Keywords:** ascidiate zone, carpel, development, evolution, flower, heterochrony, monocots, ontogeny, plicate zone, primordium

## Abstract

The phenomenon of heterochrony, or shifts in the relative timing of ontogenetic events, is important for understanding many aspects of plant evolution, including applied issues such as crop yield. In this paper, we review heterochronic shifts in the evolution of an important floral organ, the carpel. The carpels, being ovule-bearing organs, facilitate fertilisation, seed, and fruit formation. It is the carpel that provides the key character of flowering plants, angiospermy. In many angiosperms, a carpel has two zones: proximal ascidiate and distal plicate. When carpels are free (apocarpous gynoecium), the plicate zone has a ventral slit where carpel margins meet and fuse during ontogeny; the ascidiate zone is sac-like from inception and has no ventral slit. When carpels are united in a syncarpous gynoecium, a synascidiate zone has as many locules as carpels, whereas a symplicate zone is unilocular, at least early in ontogeny. In ontogeny, either the (syn)ascidiate or (sym)plicate zone is first to initiate. The two developmental patterns are called early and late peltation, respectively. In extreme cases, either the (sym)plicate or (syn)ascidiate zone is completely lacking. Here, we discuss the diversity of carpel structure and development in a well-defined clade of angiosperms, the monocotyledons. We conclude that the common ancestor of monocots had carpels with both zones and late peltation. This result was found irrespective of the use of the plastid or nuclear phylogeny. Early peltation generally correlates with ovules belonging to the (syn)ascidiate zone, whereas late peltation is found mostly in monocots with a fertile (sym)plicate zone.

## 1. Introduction

The phenomenon of heterochrony, or shifts in the relative timing, rate or duration of developmental events, is important for understanding many aspects of plant evolution, including applied issues such as crop yield [1,2,3,4,5]. In the present paper, we deal with heterochronic shifts in the evolution of an important floral organ, the carpel. Takhtajan [6] paid attention to the possible role of heterochrony (in the form that he called neoteny) in the origin of the angiosperm carpel, but angiosperm carpels are rather diverse, and the evolutionary formation of this diversity should be understood through the evolution of ontogenetic trajectories.

The carpels, being ovule-bearing organs, facilitate fertilisation, seed, and fruit formation in wild and cultivated flowering plants. It is the carpel that provides the key character of flowering plants: angiospermy. All the carpels of a flower together form a gynoecium. The carpel is not only the most important angiosperm-specific feature but also the most enigmatic one. Exact carpel homologies are unknown [7,8,9,10,11]. It is mainly for purposes of descriptive simplicity that the carpel may be considered a specialised leaf homologue (megasporophyll), even though no reliable fossil prototype can be provided to support this interpretation.

**Figure 1:**
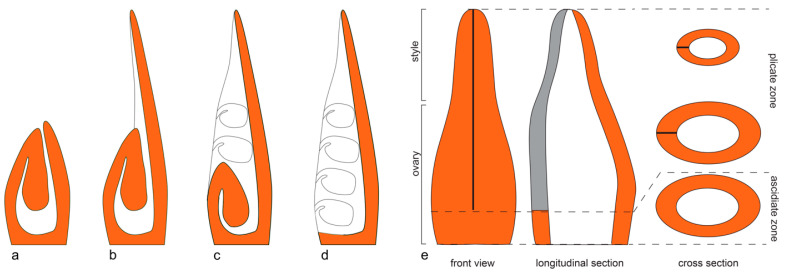
Basic carpel morphology. (**a**–**e**) Longitudinal schematic sections of different carpel types. Carpel margins with ovules attached in the plicate zone (**c**,**d**) are not in the section plane; these ovules are uncoloured here. (**a**) Completely ascidiate carpel (with no plicate zone). (**b**) Carpel with fertile ascidiate and sterile plicate zones. (**c**) Carpel with fertile ascidiate and plicate zones. (**d**) Plicate carpel (with no ascidiate zone). (**e**) Carpel with ascidiate and plicate zones as seen in frontal view, longitudinal section and a series of transversal sections. Grey in the longitudinal section shows the area of postgenital closure of the ventral slit. Ovules are not shown in (**e**). The dashed lines indicate the borders of the ascidiate and the plicate zone.

The angiosperm carpels are diverse in structure and development [12,13,14,15]. Three carpel types can be recognised (Figure 1). Their differences can be better understood in so-called apocarpous gynoecia, where carpels are free from each other or, when fused, the fusion between the carpels is postgenital. Postgenital fusion is a kind of fusion that can be directly observed as a process in ontogeny [16,17].

(1) *Completely ascidiate carpels* (Figure 1a) develop in ontogeny as sac-like structures with ovule(s) attached to the inner surface of the sac. The young ascidiate carpel has an orifice at the top. It may remain open at anthesis (usually filled with mucilage) or becomes closed by postgenital fusion of the distal margins of the carpel.
(2) *Completely plicate carpels* (Figure 1d) of free-carpellate gynoecia have a ventral slit that extends downwards up to the very base of a carpel. The ventral slit is the area of postgenital fusion of the carpel margins.
(3) *Carpels with plicate and ascidiate zones* (Figure 1b,c,e) possess a proximal part lacking a ventral slit (called the ascidiate zone) and a distal part bearing a ventral slit (called the plicate zone).

Many angiosperms possess so-called syncarpous gynoecia, in which carpels are congenitally united with each other. The term congenital fusion is a kind of fusion that cannot be directly observed in ontology but is being revealed through a comparative analysis [16,17].

When completely ascidiate carpels form a syncarpous gynoecium, its ovary has as many locules as carpels. A syncarpous gynoecium formed by completely plicate carpels is unilocular early in ontogeny because adjacent margins of neighbouring carpels are congenitally fused with each other to form a locule that is shared by all carpels. When carpels with plicate and ascidiate zones form a syncarpous gynoecium, there is a proximal portion with as many locules as carpels (called the synascidiate zone); the distal part of the young gynoecium is unilocular (called the symplicate zone). Late in ontogeny, the symplicate zone can subdivide into individual locules by ventral slit formation in each carpel.

The carpel type with plicate and ascidiate zones is rather common among angiosperms. Most studies of angiosperm gynoecia focused on the relative size and fertility of the two carpel zones in various taxonomic groups. For example, in some carpels, the plicate zone is long and fertile (i.e., bear ovules), whereas the ascidiate zone is short and sterile. In other carpels, the plicate zone is short and sterile, whereas the ascidiate zone is long and fertile. Some carpels have both zones being fertile. In some instances, there is only one ovule attached at the border between the plicate and ascidiate zones (called the cross-zone).

In the present paper, we pay attention to another aspect of carpel diversity, namely, in the relative timing of the initiation of plicate and ascidiate zones [13,18]. In carpels with plicate and ascidiate zones, the two zones do not initiate simultaneously in ontogeny. There is an option of carpel initiation with a plicate zone, with the subsequent appearance of an ascidiate zone through growth processes below it. Alternatively, the ascidiate zone can initiate first, with the subsequent appearance of a plicate zone through predominant apical growth at the dorsal side of the carpel.

We discuss patterns of carpel structure and development across monocots, a species-rich and economically important angiosperm clade. Earlier large-scale studies did not provide detailed data on global patterns of carpel evolution across monocots [19,20,21,22,23]. Relatively few studies specifically focused on the evolution of monocot flowers [23,24,25,26], apparently because the monocots are considered a derived lineage with more or less uniform flowers. Indeed, the diversity of floral constructions is much lower in monocots than in eudicots and basal angiosperms [23,24,25]. For example, the flowers of the monocotyledons are exclusively whorled and are usually trimerous pentacyclic with fused carpels. Difficulties with reconstructing the evolution of gynoecium characters in monocots are not only due to low interest. The knowledge of gynoecium construction across monocots is insufficient. On the other hand, the monocot gynoecia themselves are quite complicated. The presence and mode of carpel fusion are often poorly understood without a developmental study or homology assessment [27]. Even the carpel number is not always obvious.

For the present study, we focused on carpel characters that can be scored irrespective of fusion between carpels. Using published phylogenies [28,29,30,31,32,33,34,35,36,37,38,39] and developmental data with a few original additions, we explore the following carpel characters, distribution within monocot families, evolutionary patterns, and potential correlations: (1) the presence or absence of plicate or ascidiate carpel zones; (2) zone fertility; and (3) the sequence of carpel zone initiation during carpel ontogeny.

## 2. Results and Discussion

**Figure 2:**
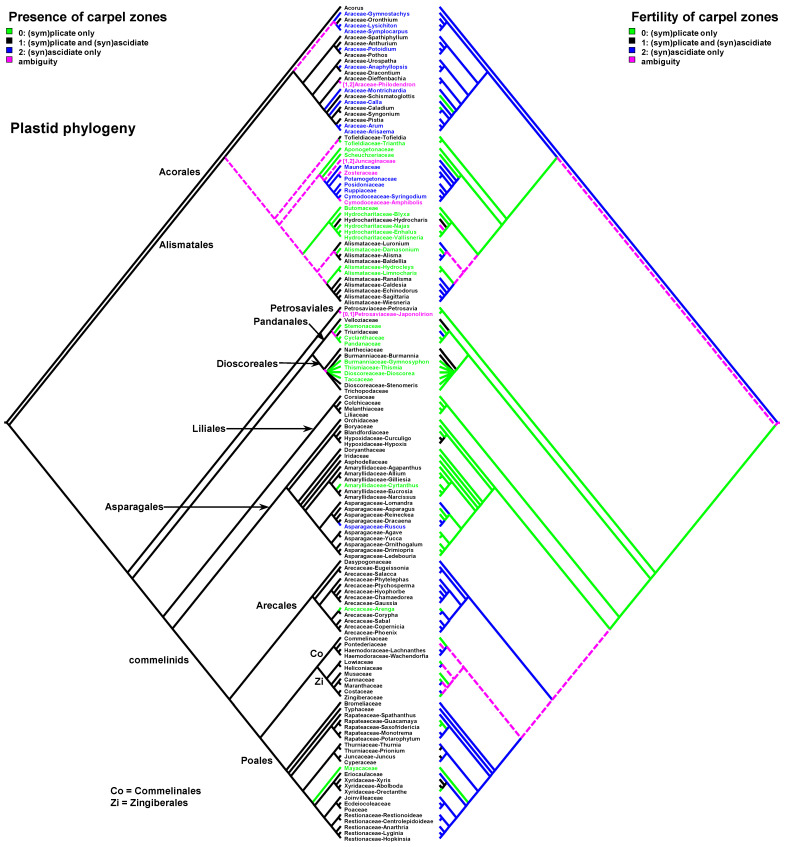
Parsimony reconstructions of the evolution of the presence of carpel zones (**left** tree and colouring of terminal groups) and fertility of carpel zones (**right** tree). Plastid phylogeny is used (see Section 3 Materials and Methods). When only one carpel zone is present, then it is, of course, fertile. When two carpel zones are present, either one of them of both can be fertile.

**Figure 3:**
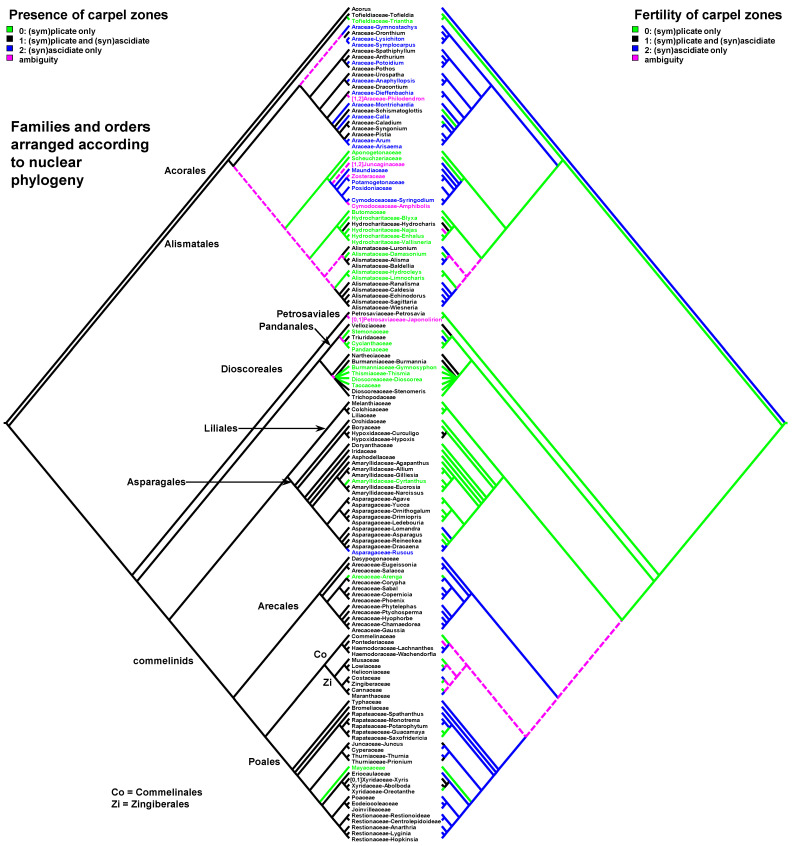
Parsimony reconstructions of the evolution of the presence of carpel zones (**left** tree and colouring of terminal groups) and fertility of carpel zones (**right** tree). Families and orders are arranged according to nuclear phylogeny [39]; otherwise, plastid phylogeny is used because of the lack of detailed nuclear data.

**Figure 4:**
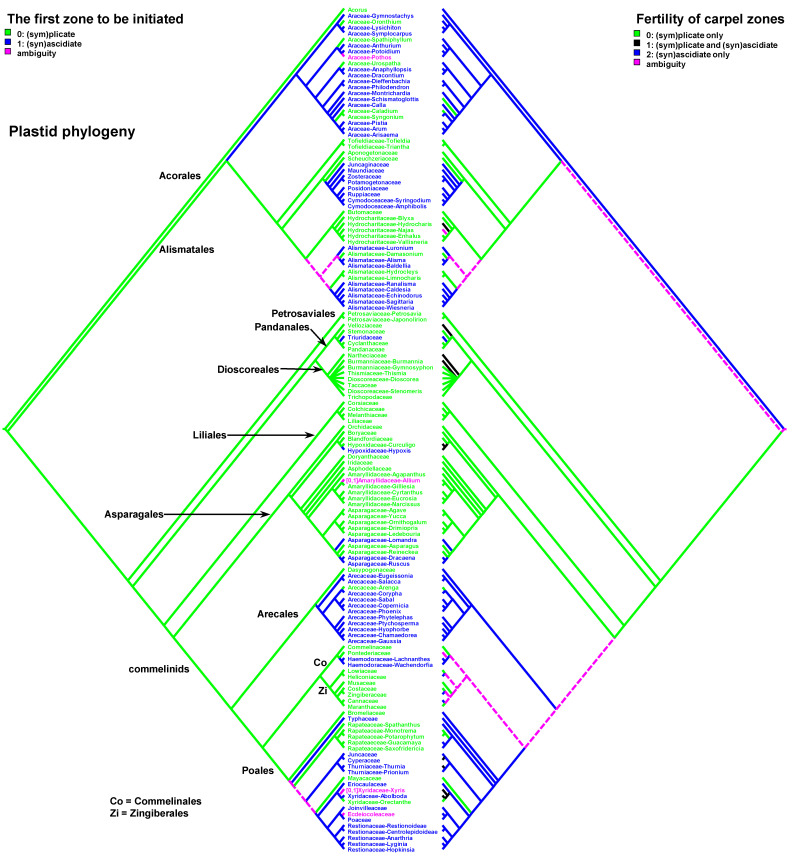
Parsimony reconstructions of the evolution of the first carpel zone to be initiated in ontogeny (**left** tree and colouring of terminal groups) and fertility of carpel zones (**right** tree). Plastid phylogeny is used (see Section 3 Materials and Methods). See Figure 5 and Figure 6 for enlarged details of relationships in Alismatales and commelinid monocots, respectively.

**Figure 5:**
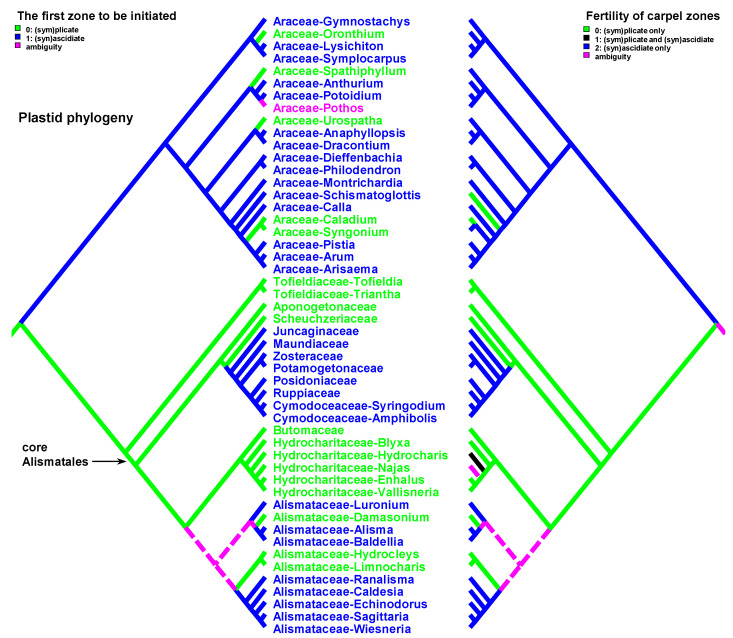
Detail of Figure 4 showing relationships in Alismatales.

**Figure 6:**
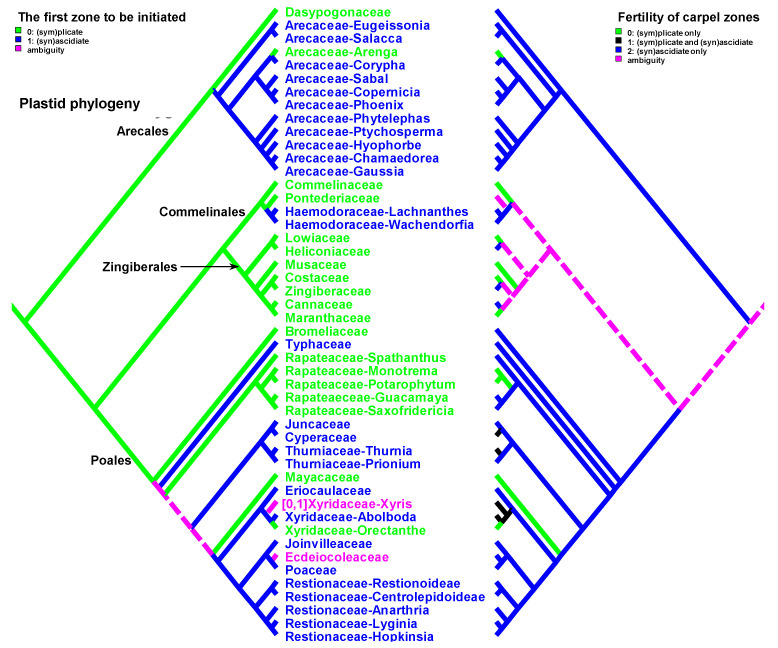
Detail of Figure 4 showing relationships in commelinid monocots.

**Figure 7:**
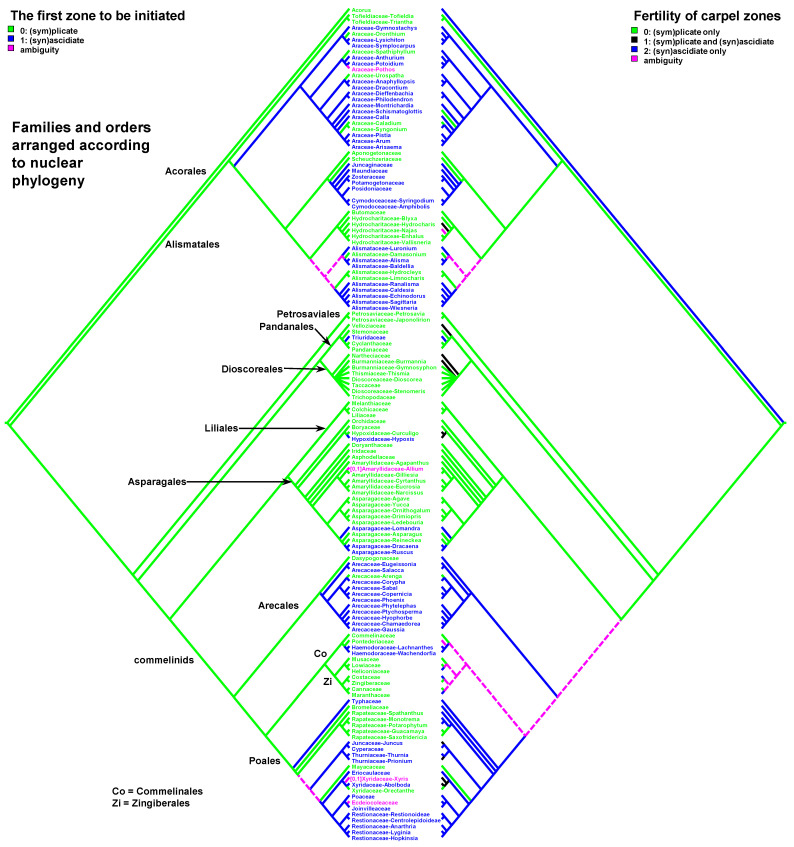
Parsimony reconstructions of the evolution of the first carpel zone to be initiated in ontogeny (**left** tree and colouring of terminal groups) and fertility of carpel zones (**right** tree). Families and orders are arranged according to nuclear phylogeny [39]; otherwise, plastid phylogeny is used because of the lack of detailed nuclear data.

Data on carpel morphology and sequence of carpel zone initiation are given in Table 1. Our parsimony reconstructions of the evolution of the three carpel characters in monocots are provided in Figure 2, Figure 3, Figure 4, Figure 5, Figure 6 and Figure 7. These analyses are based on the data summarised in Table 1.

In most cases, we were able to score characters unequivocally (Table 1). Although, there are some taxa (often with unusual flower groundplan and specialised pollination syndromes) in which the gynoecium construction is ambiguous and depends on interpretation. Such problematic cases are already present within the early divergent family Araceae. There is a long history of debates about genera of Araceae with unilocular ovary and several ovules on the basal or apical placenta [40,41]. Developmental studies and studies on gynoecium vasculature have failed to resolve questions on the carpel number involved in gynoecium formation in these genera. There are no distinct free carpel tips in Araceae either developmentally or in the mature gynoecia. In all such cases, monomerous vs pseudomonomerous (i.e., having at least two carpels) interpretation affects scoring carpel type dramatically.

In contrast to Araceae, nearly all members of the predominantly wind-pollinated Cyperaceae and Poaceae (Poales) demonstrate superficially similar gynoecia resulting from different processes of evolutionary reduction. In both families, there are difficulties with gynoecium interpretation using standard descriptive terminology. The gynoecia of grasses and sedges, like those of some eudicots, are too integrated and too reduced to recognise (sym)plicate and (syn)ascidiate zones unequivocally [42,43]. In Cyperaceae, the gynoecium has a unilocular ovary with a single basal ovule and a style with two or three stigmatic branches. Comparison with closely related Juncaceae (especially *Lusula*) suggests that the stigmatic branches correspond to free carpel tips, the style and the ovary wall represent congenitally united plicate carpel parts (symplicate zone), the placenta belongs to the reduced synascidiate zone, and the ovule is shared between the carpels being attached in the centre of conjoined septae. Thus, we scored the carpels of Cyperaceae as having both plicate and ascidiate zones.

Grasses demonstrate a gynoecium that is superficially similar to that in sedges. Apart from the rare genera apparently possessing only one carpel (e.g., *Nardus*, *Anomochloa*), the grass gynoecium has the same parts as in sedges. Despite these similarities, the nature of the ovary locule is different. A typical bistigmatic grass gynoecium has three carpels: two sterile carpels with no locules but each producing a stigma, and a fertile carpel that lacks a stigma and develops an ovule attached at the cross-zone [43,44,45]. Thus, the ovary wall corresponds to a synascidiate zone rather than a symplicate one, and the ovule is not shared by all the carpels as in sedges but belongs to the only fertile carpel. It is unclear whether the occurrence of a symplicate zone can be explicitly demonstrated in grasses, but the solid grass stigmas are apparently derived from plicate stigmas of the type found in less specialised families of Poales such as Restionaceae [43,44,46].

### 2.1. Plicate Carpels

Entirely plicate carpels are extremely rare in monocots and are found only within those families where carpels of other representatives have both zones, and the ascidiate zone, as a rule, is sterile (Table 1 and Figure 2 and Figure 3).

Carpels without an ascidiate zone are common in the order Alismatales: *Triantha* and *Pleea* (Tofieldiaceae; no developmental data for *Pleea*), monogeneric Butomaceae, Scheuchzeriaceae and Aponogetonaceae, the majority of Hydrocharitaceae included in the present analysis and some Alismataceae (especially members of the former Limnocharitaceae). Completely plicate carpels are more often found in so-called petaloid alismatids (Butomaceae, Hydrocharitaceae, and Alismataceae, Figure 2 and Figure 3). According to Kaul [47], they represent a derived condition here as evidenced by their laminar placentation (a rare condition among monocots) and complex vascular patterns. Our parsimony analyses suggest that the common ancestor of Alismatales had carpels with ascidiate and plicate zones (Figure 2 and Figure 3). In some Alismatales (e.g., in *Aponogeton*), carpels are united at their bases in the gynoecium centre (but not through carpel lateral margins), whereas their free parts are completely plicate. This causes a certain instability in the interpretation of the gynoecium and carpel construction. If interpreted as obliquely inserted on a convex receptacle [41,48] the carpels are completely free and plicate (united via the floral centre, i.e., the receptacle). Alternatively, if the receptacle is regarded as flat, the area along which the carpels are united should be regarded as a synascidiate zone. We follow the first interpretation.

Completely plicate carpels are present in *Thismia* and *Gymnosiphon* (Burmanniaceae, Dioscoreales), *Tacca* (Dioscoreaceae, Dioscoreales), all members of Stemonaceae, Cyclanthaceae and Pandanaceae (Pandanales), at least some Arecaceae, in the sole genus of Mayacaceae (Poales) and can be sporadically found in Liliales (*Scoliopus*, no developmental data available) and Asparagales (Figure 2 and Figure 3). Both Liliales and Asparagales are studied insufficiently in terms of developmental data.

In *Japonolirion* (Petrosaviaceae, Petrosaviales), the presence of an ascidiate carpel zone is variable. Examined species of two closely related genera of Tofieldiaceae (Alismatales) differ in the presence (*Tofieldia*) or absence (*Triantha*, *Pleea*) of an ascidiate carpel zone. Despite belonging to different orders, Tofieldiaceae and *Japonolirion* share many common features [23,49,50,51]. In both genera, carpels are stalked with infralocular septal nectaries developed on the ventral and lateral surfaces of the carpel stalks. Carpels are postgenitally united along the length of the ovary, but carpel stalks and styles are free.

### 2.2. Completely Ascidiate Carpels

Undoubtful ascidiate carpels without a plicate zone are an exclusive feature of some Araceae and tepaloid Alismatales—Juncaginaceae, Maundiaceae, Zosteraceae, Potamogetonaceae (including former Zannicheliaceae), Posidoniaceae, Ruppiaceae, Cymodoceaceae, whose members are either wind-pollinated helophytes or submerged fresh-water or marine plants with underwater pollination (Figure 2 and Figure 3). The mature carpels are diverse. In some Juncaginaceae, the stigma is extended around more or less vertical ventral carpel mouth. This upper part of the carpel could be regarded as a short plicate zone. The same phenomenon, though in a less pronounced form, can be found in *Maundia* and some species of *Potamogeon*. In *Ruppia*, *Posidonia* and former Zannicheliaceae, the stigma is funnel-shaped. In Zosteraceae and Cymodoceaceae, there are two or three stigmatic branches. Members of Zosteraceae always possess two lateral stigmas. The carpel apex of many early-divergent monocots is extended into two short lateral tips [41]. These tips can be regarded as extremely developed in Zosteraceae and homologised with carpel apices of other alismatids that demonstrate this feature. A carpel apex with two lateral tips is more common for early divergent monocots whose carpels possess a plicate zone (the very tip is split dorsally). In this respect, those tips in otherwise ascidiate carpels should be treated as an unusual plicate zone. However, such strongly bilobed apex has never been interpreted as a plicate zone in otherwise ascidiate carpels of the basal angiosperm *Austrobaileya* (Austrobaileyales) and in monocots [41,52]. The monocarpellary interpretation is somewhat difficult in some Cymodeceaceae due to the occurrence of three rather than two stigmas. The occurrence of three stigmas may indicate that the gynoecium of Cymodoceaceae should be treated as pseudomonomerous and consisting of two or three carpels [41].

In angiosperms, typical ascidiate carpels of ancestral type have a sessile stigma and an ovule hanging from the upper ventral carpel margin—a cross-zone [20,21,52]. The carpel mouth is sealed by secretion and usually covered by stigmatic papillae [12,53,54,55,56,57]. Such typical carpels of basal angiosperms develop as tubular structures, and the ovules (sometimes more than 1 per carpel) are initiated after the carpel walls are more or less fully formed. The ovules are not visible from the outside during carpel ontogeny (“early carpel closure”, as defined by Endress [57]). Typical ascidiate carpels with late ovule initiation and the orifice sealed by secretion are known in monocotyledons only in Araceae if the gynoecium is interpreted as monomerous [40].

Ascidiate carpels of core Alismatales are quite different from the type found in basal angiosperms. They demonstrate another construction and developmental pattern. There is a style (a sterile portion above the placenta) and a postgenital closure of the carpel orifice. In development, the ovules appear very soon after the young carpel becomes cup-shaped. The single ovule can be seen in early development before carpel walls begin their elongation and before the carpel closure. After the ovule is initiated, the carpel grows as a tube, i.e., the style seems to be formed by an ascidiate zone. This developmental behaviour of the carpel wall can be interpreted in two different ways. The first explanation implies a heterochrony in ovule initiation. The second hypothesis deals with the re-interpretation of carpel morphology. If we assume that the position of the ovule is strictly marginal, i.e., that the upper ventral carpel margin is used to form the placenta, then we have to recognise that a secondary carpel margin is formed above the ovule. The formation of a secondary margin above the cross-zone is known in some magnoliids and eudicots [13,57,58], but never reaches the same expression as in alismatids. During development, the carpel walls enclose the ovule on all sides, including the cross-zone. Thus, the carpel wall has a combined nature with a secondary ventral carpel margin above the ovule insertion and plicate zone in the other half of the carpel circumference. Due to the presence of a secondary carpel margin, the ventral slit is short and displaced at the top of the carpel. The apparent border of the ascidiate zone appears to be higher than its true (original) border. Free carpel margins at the carpel top retain their ability to postgenital fusion. Postgenital carpel closure rather than its sealing by secretion, results in more secure isolation of the carpel interior from the external environment. Presumably, this evolutionary transformation is adaptive for submerged or partly submerged habitats.

### 2.3. Carpels with Ascidiate and Plicate Zones

The vast majority of monocots possess carpels with ascidiate and plicate zones [20,21,58,59,60]. They can be classified into three subtypes—carpels with a sterile plicate zone and a fertile ascidiate zone, carpels with a fertile plicate zone and a sterile ascidiate zone and carpels with both zones producing ovules (Figure 2 and Figure 3). Carpels of the first subtype are quite common in taxa with free-carpellate gynoecia such as the majority of Alismataceae (Alismatales), Triuridaceae (Pandanales) and some palms (Arecaceae). These carpels contain a single ventral ovule and a hollow or solid plicate style. Alismataceae and Triuridaceae demonstrate early ovule initiation on the ventral side of a circular young carpel. Among taxa with syncarpy, fertile (syn)ascidiate zone with single pendent ovule is characteristic for some Asparagaceae (Asparagales), Dasypogonaceae (Arecales), some palms (Arecaceae), members of Pontederiaceae and Haemodoraceae (Commelinales), Heliconiaceae and Marantaceae (Zingiberales), *Sparganium* (Typhaceae, Poales), Rapateoideae and Monotremoideae (Rapateaceae, Poales), Eriocaulaceae and the majority of wind-pollinated Poales with trilocular ovaries. Multiple ovules associated with the ascidiate carpel zone are found in *Acorus* (Acoraceae, Acorales) and in some species of *Xyris* (Xyridaceae, Poales). In both genera, the ovules are orthotropous [41,61,62]. In *Acorus*, the placentae are hanging from the cross-zone into the ovary locules. In *Xyris*, the ovules have their micropyles facing the style, they are attached more or less basally and occupy the upper surface of an area corresponding to the fused septae in the gynoecium centre (placentation is columnar in some species) [63,64,65,66]. In general, carpels with fertile (syn)ascidiate zone are characteristically rare in the grade of lilioid monocots (Figure 2 and Figure 3).

Carpels with both zones being fertile develop U-shaped or Y-shaped marginal placentae with more or less numerous ovules. Such carpels are relatively rare and can be found in Nartheciaceae (Dioscoreales), *Burmannia* (Burmanniaceae, Burmanniales), *Velloziaceae* (Pandanales), Hypoxidaceae (Asparagales), some *Pontederia* (Pontederiaceae, Commelinales), Bromeliaceae (Poales), *Juncus* (Juncaceae, Poales and in *Abolboda* and some species of *Xyris* (Xyridaceae, Poales). This condition is morphologically very close to the carpels with ovules attached only in the plicate zone. Apparently, the number of genera with U-shaped or Y-shaped placentae is higher than it appears in the literature. The ovules attached in the cross-zone can be easily overlooked if the plicate zone is long and bears numerous ovules. In our opinion, the fertility of the ascidiate carpel zone depends on its depth. In carpels with U-shaped placentae, the ovule(s) are attached in the cross-zone (border of ascidiate and plicate zones) and hang down into the ascidiate zone itself. In *Tofieldia* (Tofieldiaceae, Alismatales), the depth of the ascidiate zone is insufficient to accommodate the ovule and future seed, while in *Harperocallis* of the same family (no developmental data available for this genus) the ascidiate zone is very well expressed and fertile [67]. The same is true for representatives of Petrosaviaceae, where the depth of the ascidiate zone varies within the same species and often it is not deep enough to host an ovule [49,68,69].

### 2.4. Patterns of Carpel Development

The carpels with ascidiate and plicate zones can develop in two different ways with regard to the cross-zone formation during carpel development, i.e., so-called peltation [13]. In the case of primary peltation, the carpel development starts with a cup-shaped or circular primordium and has a cross-zone (ventral carpel wall) from the very beginning of development. In carpels with secondary peltation, the ascidiate zone appears later in development (see also [18]) and the carpel starts as a crescent or horseshoe-shaped primordium. The variants of secondary peltation in development are rather diverse [13], but in our opinion, they represent the same pattern with the ascidiate zone being formed by zonal growth under the plicate zone.

**Figure 8:**
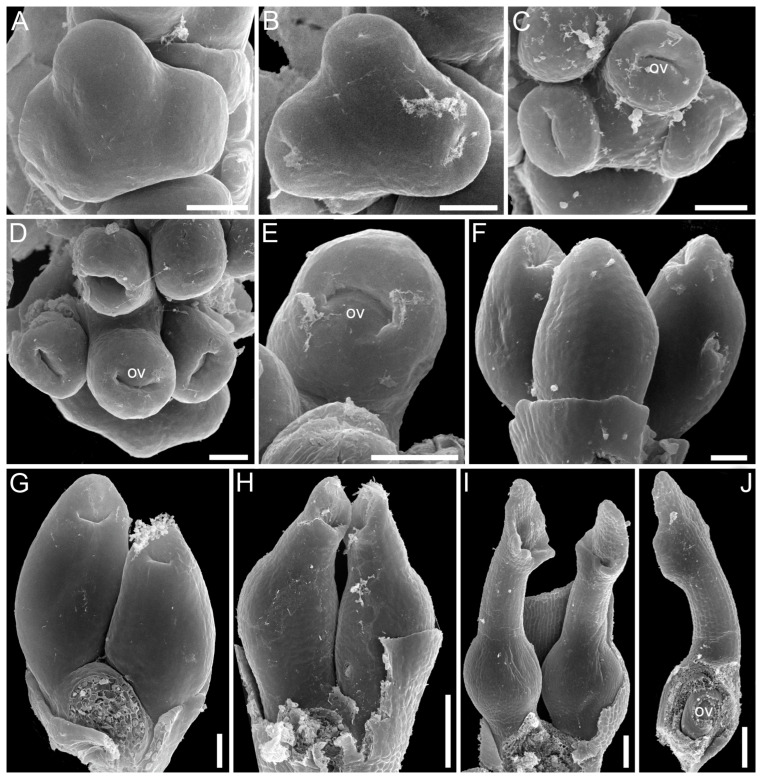
Ascidiate carpel development with early ovule initiation in *Zannichellia palustris* (Potamogetonaceae, Alismatales). (**A**) Carpel primordia before peltation. (**B**) Beginning of peltation, carpels become ring-shaped due to formation of a central depression. (**C**,**D**) Ovule initiation at the ventral carpel margin. (**E**) Beginning of carpel wall growth above the ovule, the growth starts on the dorsal carpel side. (**F**–**H**) Subsequent elongation of carpel wall, the carpel develops as a tubular structure. (**I**) Mature carpel with rounded ovary, slender style and funnel-shaped stigma. (**J**) Mature carpel dissected to show single ovule. Ov, ovule. Scale bars = 30 mkm (**A**–**G**) and 100 mkm (**H**–**J**).

**Figure 9:**
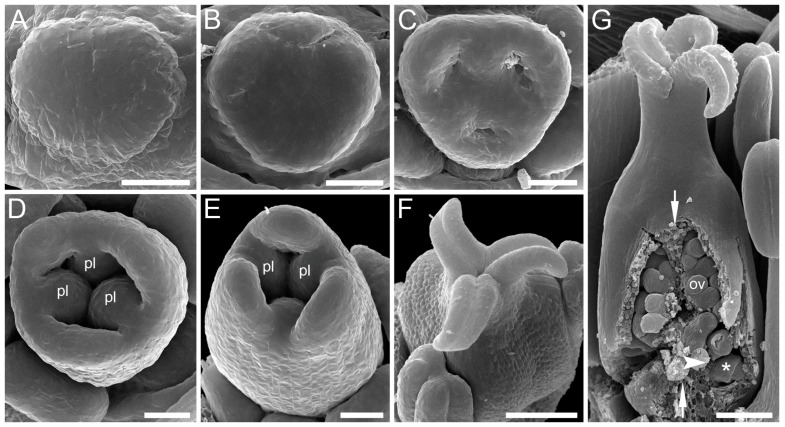
Gynoecium development in *Juncus bufonius* (Juncaceae, Poales). In *Juncus*, both carpel zones are fertile. (**A**,**B**) Gynoecium primordium before carpel initiation. (**C**) Initiation of synascidiate zone, individual carpels are seen as depressions in gynoecium corners. (**D**,**E**) Formation of symplicate zone with massive placentae; placentae correspond to congenitally united margins of neighbouring carpels in their plicate zones. (**F**) Gynoecium closure and elongation of styles. (**G**) Young gynoecium dissected to show ovules; arrows show a boundary of two neighbouring carpels; arrowhead shows a boundary of plicate and ascidiate zones in the right carpel; the ovule marked with an asterisk (*) is attached in the ascidiate zone (or cross-zone). Ov, ovule; pl, placenta. Scale bars = 30 mkm (**A**–**E**) and 100 mkm (**F**,**G**).

The sequence of carpel zone initiation is largely a matter of ovule insertion (Figure 4, Figure 5, Figure 6 and Figure 7). In most cases, in taxa with fertile ascidiate and sterile plicate zones (usually a single pendent ovule attached in the cross-zone), the ascidiate zone is first to be initiated. The plicate zone is formed later if it is present at all. Early ovule initiation [57] is very characteristic for taxa with free carpels (Figure 8) or carpels united via the floral centre [59,70,71,72,73,74] but can be found in a few genera with typical syncarpy [60]. In syncarpous gynoecia, the ascidiate zones of neighbouring carpels are ab initio united (i.e., congenitally fused), and the gynoecial synascidiate zone is initiated as the entire structure. Such a young gynoecium is more or less triangular in outlines (if there are three carpels) with tree depressions in the corners that correspond to the future ovary locules (Figure 9). In the members of the order Poales, the portion of meristematic tissue in the gynoecium centre between the locules is considerable. This bulge can serve as a placenta to accommodate additional ovules. Sometimes, this region is protruding and forms a columnar placenta as in some species of *Xyris* (Xyridaceae, Poales) [63,64,75].

**Figure 10:**
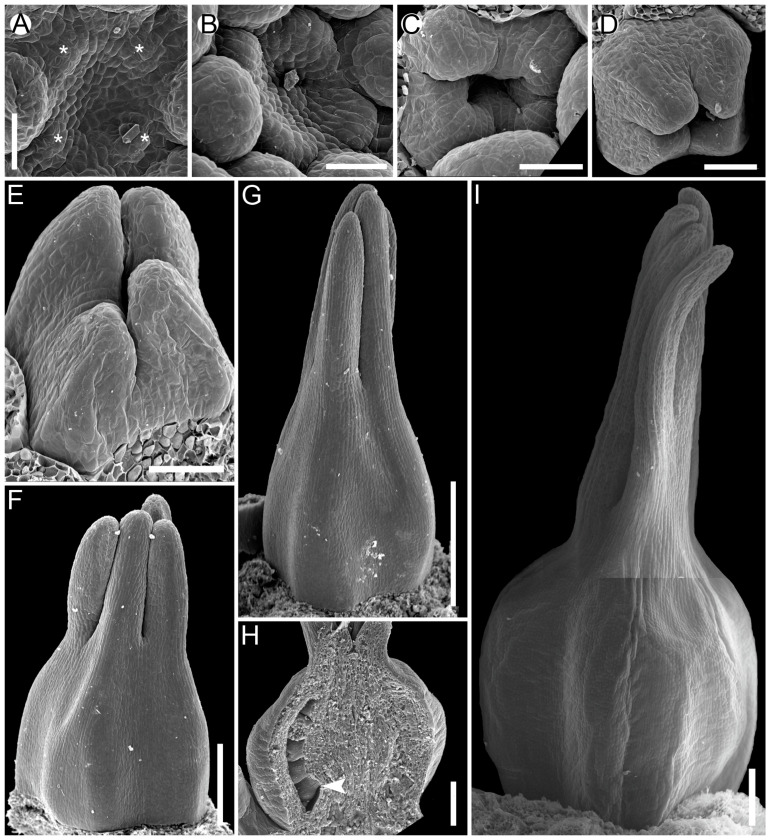
Gynoecium development in *Paris quadrifolia* (Melanthiaceae, Liliales). In *Paris*, both carpel zones are present, and the plicate zone is fertile. (**A**) Gynoecium initiation: carpels (*) are initiated as slightly crescent-shaped primordia (plicate zone). (**B**) Young gynoecium with free carpels. (**C**–**E**) Elongation of the symplicate zone under free plicate carpel parts (so-called asymplicate zone). (**F**,**G**) Further development of plicate parts of the carpels; free styles comprise the asymplicate gynoecium zone, and the ovary is formed by the symplicate zone at this stage. (**H**,**I**) Young gynoecium with both carpel zones developed; the gynoecium dissected in (**H**) to show ovule insertion; an arrowhead shows a boundary between the plicate and ascidiate zones. Scale bars = 100 mkm (**A**–**C**), 120 mkm (**D**,**E**), 400 mkm (**F**), 600 mkm (**G**), and 300 mkm (**H**,**I**).

**Figure 11:**
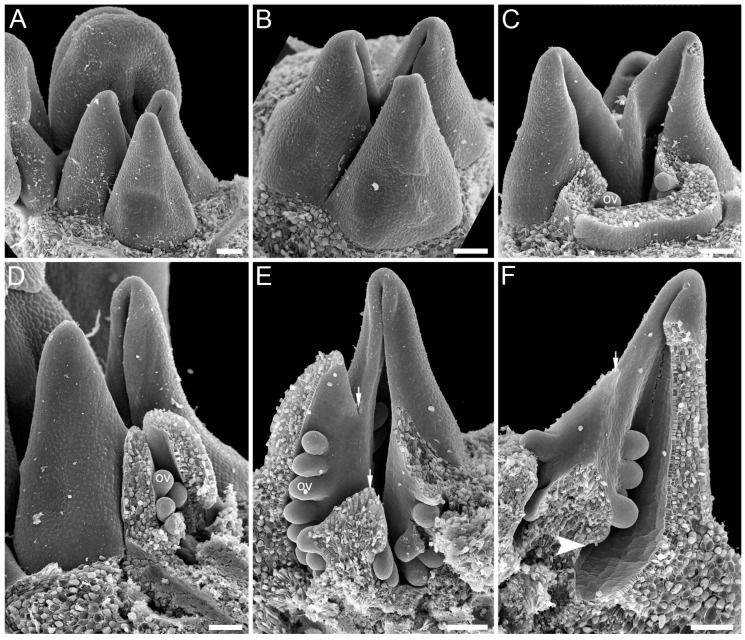
Gynoecium development in *Anticlea elegans* (Melanthiaceae, Liliales). In *Anticlea*, both carpel zones are present, and the plicate zone is fertile. (**A**,**B**) Developing gynoecium (same in both images) consisting of nearly free plicate carpels. (**C**) Slightly later stage with one carpel removed to show ovule initiation in symplicate zone (below free carpel parts). (**D**) Gynoecium with one carpel open on its dorsal side to show ovules attached along carpel margins in the plicate carpel zone. (**E**,**F**) Two parts of a dissected gynoecium; arrows show a boundary of two neighbouring carpels; an arrowhead shows a boundary between the plicate and ascidiate zones in (**F**). Ov, ovules. Scale bars = 100 mkm.

Carpels with either both zones fertile or only the plicate zone fertile usually commence their ontogeny with a plicate zone (Table 1, Figure 10 and Figure 11). Early ovule initiation has not been reported for such carpels. The ovule cannot be seen from the outside of a gynoecium without a dissection at any developmental stage.

**Figure 12:**
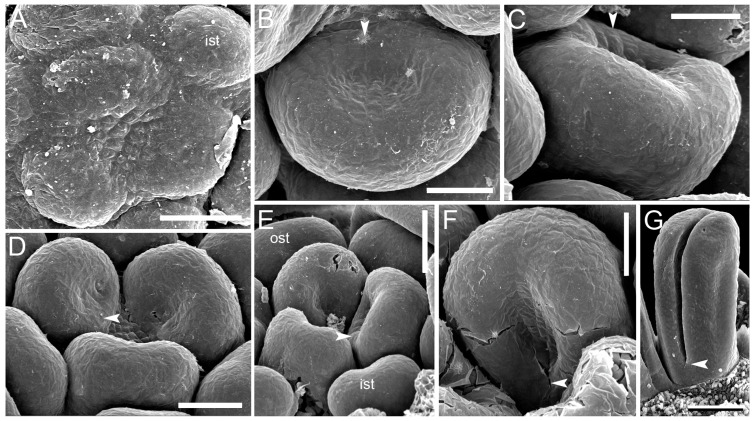
Gynoecium development in *Tofieldia coccinea* (Tofieldiaceae, Alismatales); both carpel zones are present, and the plicate zone is fertile. (**A**) Carpel initiation as slightly crescent-shaped primordia (plicate zone). (**B**,**C**) Cup-shaped carpels with peltation completed (the ventral carpel wall is already present). (**D**,**E**) Gynoecia with one carpel showing a peltation, other two carpels are still completely plicate. (**F**,**G**) Carpels at subsequent developmental stages. Arrowheads, the ventral carpel wall and the boundary of the plicate and ascidiate zones. ist, inner whorl stamen; ost, outer whorl stamen. Scale bars = 60 mkm (**A**,**D**), 40 mkm (**B**,**C**,**F**), 80 mkm (**E**) and 100 mkm (**G**).

There are some exceptions to these two basic patterns (Figure 4, Figure 5, Figure 6 and Figure 7). Most of them comprise genera and families with the late initiation of the fertile ascidiate zone, i.e., the sterile plicate zone is first to be initiated. This pattern is characteristic of *Acorus* (Acoraceae), some genera of Araceae (Alismatales), *Dasypogon* (Dasypogonaceae, Arecales), Heliconiaceae, Marantaceae and Costaceae (Zingiberales), subfamilies Rapateoideae and Monotremoideae in Rapateaceae (Poales). An alternative situation—early initiation of sterile ascidiate zone—can be found in some genera of Araceae (Alismatales) and a species of *Allium* (Amaryllidaceae) (for references see Table 1). The genus *Tofieldia* (Tofieldiaceae, Alismatales) is of particular interest because within a single species or even within the same flower carpels sometimes show different developmental patterns. In *Tofieldia*, the ascidiate zone is sterile whereas the plicate one is fertile and is first to arise but carpels show different relative timing of peltation (Figure 12). In some carpels, ventral carpel wall forms very early and such carpels are cup-shaped in their early development. The reason for such variability is not clear. In the mature gynoecium of *Tofieldia*, all carpels are identical, with a short ascidiate zone.

### 2.5. Carpel Types and Monocot Taxonomy

In general, the carpel type and the order of zone appearance seem to be conservative at least at the genus or family level. The diversity of patterns of carpel structure and development has no clear taxonomic assignment. The majority of monocot orders contain at least some representatives with contrasting carpel morphologies. The only thing that can be noticed is that in some families and orders, the carpel morphology is more stable. Thus, almost all members of the order Liliales demonstrate carpels with fertile plicate and sterile ascidiate zones, and the plicate zone is initiated before the ascidiate one. All representatives of the families Potamogetonaceae (Alismatales), Orchidaceae (Asparagales) or Eriocaulaceae (Polales) have uniform gynoecium structure within their families. Greater diversity in the carpel structure is observed in the early-divergent order Alismatales and in families occupying near-basal positions within their orders. Among Alismatales, the aroids possess the most variable carpel and gynoecium constructions. This is apparently not only due to the great number of genera. Araceae is among the most problematic monocot families in terms of gynoecium interpretation. The number of carpels involved in gynoecium formation and zone of ovule insertion are not clear for many genera [40,41]. Tofieldiaceae (another near-basal member of Alismatales) is remarkable for the variable presence of plicate zone and mode of carpel development (see above). Entirely plicate carpels are apparently apomorphic here and evolved via the reduction of the ascidiate carpel zone. Alismataceae usually develop uniovulate carpels with early ascidiate carpel development, but now Alismataceae hosts the genera of former Limnocharitaceae whose plicate carpels with laminar placentation resemble carpels of Butomaceae. Among Dioscoreales, representatives of Nartheciaceae and the genus *Burmannia* (Burmanniaceae) have carpels with both carpel zones bearing the ovules. In other genera of Dioscoreales, the ascidiate zone is fertile or even lacking. Members of Poales usually develop carpels with a single ovule per ovary locule or per the gynoecium as a whole. The fertile plicate zone is present only in Bromeliaceae and some Rapateaceae. Among Rapateaceae, representatives of the subfamily Saxofridericioideae are characterised by a short sterile ascidiate zone and long fertile plicate zone, accommodating several ovules; in representatives of the subfamilies Rapateoideae and Monotremoideae, the plicate zone is sterile, and ovules, usually one per carpel, are placed in the ascidiate zone. Other examples are listed in Table 1.

### 2.6. Carpel Evolution

By the second half of the 20th century, there were two competing hypotheses on carpel evolution. Most authors argued in favour of (condu)plicate carpel theory [6,76,77,78,79,80,81]. According to the plicate hypothesis, the ancestral carpels were free and stalked, without an ascidiate zone and contained numerous ovules on the (sub)marginal placenta. The ventral slit was closed postgenitally. The original primitive carpels were large and spirally arranged on a convex elongated receptacle. The evolution of the gynoecium and the carpel, according to these ideas is directed toward the reduction and stabilisation of the number of organs (see also [82,83]). The ascidiate carpels represent a derived type and appeared later in evolution via the replacement of postgenital fusion in the basal part of the carpel by a congenital one.

Arguments against the ‘primitiveness’ of the conduplicate carpel appeared in the second half of the 20th century [58,59,84,85,86,87]. Van Heel [58,59,88] investigated a considerable number of genera of both dicot and monocot plants with apocarpous gynoecium that had been recognised as having archaic traits. In the majority of these plants, the carpels were shown to be initiated by circular primordia and have a more or less pronounced ascidiate zone (though not always well visible without anatomical examination at maturity). As pointed out by van Heel, the carpel stalk develops only in the presence of the ascidiate zone. Based on the developmental data, van Heel concluded that completely plicate carpels are very rare and have evolved from carpels to have both plicate and ascidiate zones. Mapping of carpel characters onto molecular-phylogenetic trees supports the ancestral condition for ascidiate carpels (e.g., [8,21,54]). According to these ideas, ascidiate carpels are ancestrally small, with a single ovule attached in the cross-zone, i.e., on the ventral side at the border of the ascidiate and plicate zones if the latter is present. According to the ‘ascidiate hypothesis’, it can be assumed that the plicate zone appeared in the evolution due to the need to accommodate a larger number of ovules via unequal growth of the dorsal carpel wall and vertical elongation of the carpel mouth [8,21,54]. The well-developed plicate zone is viewed as an important feature of the clade of mesangiosperms, even though it is absent in some members of the group [57]. Postgenital fusion of carpel margins represents a derived condition in the framework of this hypothesis. Postgenital fusion is a complex process involving dedifferentiation and redifferentiation of contacting epidermal layers. It is mostly known in angiosperms and is most commonly associated with carpel closure or fusion between the carpels in the plicate zone [8,16,53].

Both hypotheses describe general ideas on carpel evolution in angiosperms as a whole. By varying the length of the ascidiate and plicate zones up to the complete absence of one of them, it is possible to obtain a complete set of angiosperm carpel morphologies. Nevertheless, the ancestral carpel type for monocots is vague. The ascidiate carpels with a single ovule occur only in monocots with apocarpous gynoecia. Free carpels represent an evolutionary derived condition in monocots [19,20,21,23]. Endress and Doyle [20,21] concluded that the ancestral condition for monocots is a carpel with a fertile plicate zone but their matrix includes only a limited number of monocot taxa. Sauquet et al. [19] did not score this character, their matrix included only the number of ovules per carpel. Our data strongly suggest that the common ancestor of monocots had carpels with both zones (ascidiate and plicate) and late peltation in ontogeny (the plicate zone was first to initiate). What carpel zone(s) was ancestrally fertile in monocots remains unclear, partly because of the unusual gynoecium morphology of the sister to all other extant monocots, *Acorus.*

The present study provides results of the use of a top-down approach in search of the ancestral character states of the monocot carpel [89]. Monocots is a well-defined monophyletic unit. Their potential extant sister groups are relatively distantly related and morphologically diverse. Monocots form one of the five clades that belong to the group of mesangiosperms, but details of relationships among these clades are still somewhat unresolved. Gynoecium and carpel interpretation of two mesangiosperm clades, Ceratophyllaceae and Chloranthaceae, is ambivalent [90]. Therefore, the use of the top-down approach for monocots is reasonable.

**Table 1:** Carpel structure and sequence of zone initiation in monocot genera. Only genera with known carpel ontogeny are incuded. References are mostly focused of studies supported by scanning electron microscopy, where possible. !, taxa with a unilocular multicarpellate gynoecium and a single ovule. *, taxa with early ovule initiation in an ascidiate carpel zone. #, taxa with a unicarpellate or pseudomonomerous gynoecium, depending on an interpretation (in each instance, an upper row shows the conditions implied if the gynoecium is interpreted as unicarpellate, and a lower row if treated as pseudomonomerous). ?, instances where additional research is necessary for a precise decision. Zone fertility and its early initiation (before the other zone) are shown by the same colour (green for the plicate zone and orange for the ascidiate zone). Red shows cases when zone fertility and its early initiation do not match each other. Numbers in brackets after the names of the families correspond to the accepted number of genera (after POWO, https://powo.science.kew.org/, accessed 1 August 2023).

Family	Genus	Plicate Zone	Ascidiate Zone	First Zone to Be Initiated	Notes	References
**ACORALES**						
Acoracerae (1)	*Acorus*	+	+fertile	plicate		[61,72,91]
**ALISMATALES**						
Araceae (143)	*Anaphyllopsis* # (monomery)	−	+fertile	ascidiate	unilocular with 1–2 basal ovules	[92]
	*Anaphyllopsis* # (pseudomonomery)	+	+?reduced	plicate		
	*Arum*	−	+fertile	ascidiate	unicarpellate?	[93]
	*Anthurium*	+	+fertile	ascidiate		[94,95]
	*Arisaema* # (monomery)	−	+fertile	ascidiate	unilocular with several basal ovules	[96]
	*Arisaema* # (pseudomonomery)	+	+?reduced	plicate		
	*Caladium*	+fertile	+	plicate		[97]
	*Calla* # (monomery)	−	+fertile	ascidiate	unilocular with several basal ovules	[98] [99]
	*Calla* # (pseudomonomery)	+	+?reduced	plicate		
	*Dieffenbachia*	+	+fertile	ascidiate		[100]
	*Dracontium*	+	+fertile	ascidiate		[101]
	*Gymnostachys*	−	+fertile	ascidiate	unicarpellate?	[40]
	*Lysichiton*	−	+fertile	ascidiate	1−2 carpels	[40]
	*Montrichardia* # (monomery)	−	+fertile	ascidiate	unilocular with 1–2 basal ovules	[102,103]
	*Montrichardia* # (pseudomonomery)	+	+?reduced	plicate		
	*Oronthium*	+	+fertile	plicate	unicarpellate?	[40]
	*Philodendron*	+/−	+fertile	ascidiate		[104,105,106,107,108,109]
	*Pistia*	+	+fertile	ascidiate	unicarpellate?	[110]
	*Potoidium*	−	+fertile	ascidiate	unicarpellate?	[40]
	*Pothos*	+	+fertile	?		[40]
	*Schismatoglottis*	+fertile	+	ascidiate		[111]
	*Spathiphyllum*	+	+fertile	plicate		[40]
	*Symplocarpus*	−	+fertile	ascidiate	unicarpellate?	[112]
	*Syngonium*	+	+fertile	plicate		[113]
	*Urospatha*	+	+fertile	plicate		[114]
Tofieldiaceae (4)	*Tofieldia*	+fertile	+	plicate		[17,49,60,115]
	*Triantha*	+fertile	−	plicate		Unpubl. data
Alismataceae (18)	*Alisma*	+	+fertile	ascidiate *		[59,116]
	*Baldellia*	+	+fertile	ascidiate *		[117]
	*Caldesia*	+	+fertile	ascidiate *		[118,119,120]
	*Damasonium*	+fertile	−	plicate		[117]
	*Echinodorus*	+	+fertile	ascidiate *		[121]
	*Hydrocleys*	+fertile	−	plicate		[122]
	*Limnocharis*	+fertile	−	plicate		[123]
	*Luronium*	+	+fertile	ascidiate *		[124]
	*Ranalisma*	+	+fertile	ascidiate *		[70,125]
	*Sagittaria*	+	+fertile	ascidiate *		[73,126,127]
	*Wiesneria*	+	+fertile	ascidiate *		[128]
Butomaceae (1)	*Butomus*	+fertile	−	plicate		[59,60,117,129]
Hydrocharitaceae (14)	*Blyxa*	+fertile	−	plicate		[130]
	*Enhalus*	+fertile	−	plicate		[131]
	*Hydrocharis*	+fertile	+fertile	plicate		[132]
	*Najas* # (monomery)	−	+fertile	ascidiate		[133]
	*Najas* # (pseudomonomery)	+fertile?	−	plicate		
	*Vallisneria*	+fertile	−	plicate		[134]
Scheuchzeriaceae (1)	*Scheuchzeria*	+fertile	−	plicate		[135,136]
Aponogetonaceae (1)	*Aponogeton*	+fertile	−	plicate		[23,137]
Juncaginaceae (3)	*Triglochin*	−/+	+fertile	ascidiate *		[23,74,138,139]
Maundiaceae (1)	*Maundia*	−	+fertile	ascidiate (?*)		[140]
Zosteraceae (2)	*Phyllospadix*	+(?)	+fertile	ascidiate *	two stigmatic branches	[141]
	*Zostera*	+(?)	+fertile	ascidiate *	two stigmatic branches	[142]
Potamogetonaceae (5)	*Althenia*	−	+fertile	ascidiate *		[71]
	*Groenlandia*	−	+fertile	ascidiate *		[143]
	*Potamogeton*	−	+fertile	ascidiate *		[144,145,146,147]
	*Zannichellia*	−	+fertile	ascidiate *		[148]
Posidoniaceae (1)	*Posidonia*	−	+fertile	ascidiate *		[149]
Ruppiaceae (1)	*Ruppia*	−	+	ascidiate *		[150,151,152,153]
Cymodoceaceae (6)	*Amphibolis*	+(?)	+fertile	ascidiate *	two stigmatic branches	[154]
	*Syringodium*	+(?)	+fertile	ascidiate *	two stigmatic branches	[155]
**PETROSAVIALES**						
Petrosaviaceae (2)	*Petrosavia*	+fertile	+fertile/−	plicate		[49]
	*Japonolirion*	+fertile	−/+	plicate		[49]
**DIOSCOREALES**						
Nartheciaceae (5)	*Metanarthecium*	+fertile	+fertile	plicate		[156]
	*Narthecium*	+fertile	+fertile	plicate		[49,156]
Burmanniaceae s.l. (13)	*Burmannia*	+fertile	+fertile	plicate		[157,158,159]
	*Gymnosiphon*	+fertile	−	plicate		[158]
	*Thismia*	+fertile	−	plicate		[158,160]
Dioscoreaceae (4)	*Dioscorea*	+fertile	+	plicate		[158,161]
	*Tacca*	+fertile	−	plicate		[158]
	*Stenomeris*	+ fertile	+	plicate		[158]
	*Trichopus*	+fertile	+	plicate		[158]
**PANDANALES**						
Triuridaceae (8)	*Lacandonia*	+	+fertile	ascidiate *		[162,163,164,165]
	*Peltophyllum*	+	+fertile	ascidiate *		[163]
	*Sciaphila*	+	+fertile	ascidiate *		[163,166,167]
	*Triuridopsis*	+	+fertile	ascidiate *		[163]
	*Triuris*	+	+fertile	ascidiate *		[162,163]
Velloziaceae (6)	*Barbacenia*	+fertile	+fertile	plicate		[168]
	*Vellozia*	+fertile	+fertile	plicate		[168]
Stemonaceae (4)	*Pentastemona*	+fertile	−	plicate		[169]
	*Stemona*	+fertile	−	plicate		[169]
	*Stichoneuron*	+fertile	−	plicate		[169]
Cyclanthaceae (12)	*Carludovica*	+fertile	−	plicate		[170]
	*Cyclanthus*	+fertile	−	plicate		[171]
Pandanaceae (5)	*Freycinetia*	+fertile	−	plicate		[172,173]
**LILIALES** No data on: Campynemataceae, Petermanniaceae, Alstroemeriaceae, Philesiaceae, Ripogonaceae, Smilacaceae						
Corsiaceae (3)	*Arachnitis*	+fertile	+(?)	plicate		[174,175]
Melanthiaceae (14)	*Anticlea*	+fertile	+	plicate		This study
	*Chamaelirium*	+fertile	+	plicate		[176]
	*Paris*	+fertile	+	plicate		[177], this study
	*Trillium*	+fertile	+	plicate		[178]
	*Veratrum*	+fertile	+	plicate		[24,60]
Colchicaceae (15)	*Colchicum*	+fertile	+	plicate		[179]
	*Gloriosa*	+fertile	+	plicate		[60]
Liliaceae (15)	*Lilium*	+fertile	+	plicate		[180]
	*Tricyrtis*	+fertile	+	plicate		[23]
**ASPARAGALES** No data on: Asteliaceae, Lanariaceae, Ixioliriaceae, Tecophilaeaceae, Xeronemataceae						
Orchidaceae (705)	*Acineta*	+fertile	+	plicate		[181]
	*Acriopsis*	+fertile	+	plicate		[181]
	*Anthogonium*	+fertile	+	plicate		[181]
	*Apostasia*	+fertile	+	plicate		[182]
	*Bletia*	+fertile	+	plicate		[183,184]
	*Brachycorythis*	+fertile	+	plicate		[185]
	*Brownleea*	+fertile	+	plicate		[186]
	*Calanthe*	+fertile	+	plicate		[181,184]
	*Caleana*	+fertile	+	plicate		[184]
	*Calochilus*	+fertile	+	plicate		[184]
	*Cephalanthera*	+fertile	+	plicate		[187]
	*Coeloglossum*	+fertile	+	plicate		[183]
	*Corycium*	+fertile	+	plicate		[184]
	*Corymborkis*	+fertile	+	plicate		[184,187]
	*Cyclopogon*	+fertile	+	plicate		[184,187]
	*Cynorkis*	+fertile	+	plicate		[188]
	*Cypripedium*	+fertile	+	plicate		[184,189]
	*Dactylorhiza*	+fertile	+	plicate		[183]
	*Disa*	+fertile	+	plicate		[184,186]
	*Doritis*	+fertile	+	plicate		[181]
	*Diuris*	+fertile	+	plicate		[184]
	*Elleanthus*	+fertile	+	plicate		[181]
	*Epidendrum*	+fertile	+	plicate		[181]
	*Epipactis*	+fertile	+	plicate		[187]
	*Gennaria*	+fertile	+	plicate		[183,184]
	*Goodyera*	+fertile	+	plicate		[187]
	*Govenia*	+fertile	+	plicate		[181]
	*Habenaria*	+fertile	+	plicate		[72,91,183,188]
	*Herminium*	+fertile	+	plicate		[190]
	*Holothrix*	+fertile	+	plicate		[185]
	*Listera*	+fertile	+	plicate		[187]
	*Ludisia*	+fertile	+	plicate		[187]
	*Malaxis*	+fertile	+	plicate		[181]
	*Microtis*	+fertile	+	plicate		[183]
	*Monadenia*	+fertile	+	plicate		[186]
	*Neobenthamia*	+fertile	+	plicate		[181]
	*Neobolusia*	+fertile	+	plicate		[185]
	*Oeceoclades*	+fertile	+	plicate		[184]
	*Oncidium*	+fertile	+	plicate		[184]
	*Orchis*	+fertile	+	plicate		[183,184]
	*Orthoceras*	+fertile	+	plicate		[184]
	*Pholidota*	+fertile	+	plicate		[184]
	*Phragmipedium*	+fertile	+	plicate		[189]
	*Platanthera*	+fertile	+	plicate		[183]
	*Polystachya*	+fertile	+	plicate		[181,184]
	*Prasophyllum*	+fertile	+	plicate		[184]
	*Prescottia*	+fertile	+	plicate		[187]
	*Satyridium*	+fertile	+	plicate		[191]
	*Satyrium*	+fertile	+	plicate		[184,188,191]
	*Schizochilus*	+fertile	+	plicate		[185]
	*Schizodium*	+fertile	+	plicate		[186]
	*Selenipedium*	+fertile	+	plicate		[189]
	*Stenoglottis*	+fertile	+	plicate		[188]
	*Neuwiedia*	+fertile	+	plicate		[182]
	*Vanilla*	+fertile	+	plicate		[183]
	*Zygostates*	+fertile	+	plicate		[192]
Boryaceae (2)	*Alania*	+fertile	+	plicate		[193]
Blandfordiaceae (1)	*Blandfordia*	+fertile	+	plicate		[193]
Hypoxidaceae (5)	*Curculigo*	+fertile	+fertile	plicate		[193,194]
	*Hypoxis*	+fertile	+fertile	ascidiate		[193]
Doryanthaceae (1)	*Doryanthes*	+fertile	+	plicate		[193]
Iridaceae (69)	*Crocus*	+fertile	+	plicate		[195]
	*Freesia*	+fertile	+	plicate		[196]
	*Iris*	+fertile	+	plicate		[197]
Asphodelaceae (41)	*Bulbine*	+fertile	+	plicate		[60,198]
	*Eremurus*	+fertile	+	plicate		[199]
	*Hemerocallis*	+fertile	+	plicate		[60]
	*Kniphofia*	+fertile	+	plicate		[60]
Amaryllidaceae (71)	*Agapanthus*	+fertile	+	plicate		[200]
	*Allium*	+fertile	+	plicate/ascidiate		[60,72,91,201,202]
	*Cyrtanthus*	+fertile	−	plicate		[203]
	*Eucrosia*	+fertile	+	plicate		[204]
	*Gilliesia*	+fertile	+	plicate		[205]
	*Narcissus*	+fertile	+	plicate		[206]
Asparagaceae (121)	*Agave*	+fertile	+	plicate		[207]
	*Asparagus*	+fertile	+	plicate		[208,209,210]
	*Dracaena*	+	+fertile	ascidiate *		[60]
	*Drimiopsis*	+fertile	+	plicate		This study
	*Ledebouria*	+fertile	+	plicate		[23,72,91,211]
	*Lomandra*	+	+fertile	ascidiate *		[212]
	*Ornithogalum*	+fertile	+	plicate		[60,213]
	*Reineckea*	+fertile	+	plicate		[214]
	*Ruscus* # (monomery)	−	+	ascidiate		[72,91,215]
	*Ruscus* # (pseudomonomery)	+fertile	+	plicate		
	*Yucca*	+fertile	+	plicate		Unpubl. data
**ARECALES**						
Dasypogonaceae (4)	*Dasypogon*	+	+fertile	plicate		[216]
Arecaceae (182)	*Arenga*	+fertile	−	plicate		[60]
	*Chamaedorea*	+	+fertile	ascidiate *		[217]
	*Copernicia*	+	+fertile	ascidiate *		[218]
	*Corypha*	+	+fertile	ascidiate		[218]
	*Eugeissonia*	+	+fertile	ascidiate		[219]
	*Gaussia*	+	+fertile	ascidiate *		[220]
	*Hyophorbe*	+	+fertile	ascidiate *		[217]
	*Palandra*	+	+fertile	ascidiate		[219]
	*Phoenix*	+	+fertile	ascidiate *		[221]
	*Ptychosperma*	+	+fertile	ascidiate *		[222]
	*Sabal*	+	+fertile	ascidiate		[218]
	*Salacca*	+	+fertile	ascidiate *		[60]
**COMMELINALES** No data on: Hanguanaceae, Philydraceae						
Commelinaceae (36)	*Callisia*	+fertile	+	plicate		[223]
	*Cochliostema*	+fertile	+	plicate		[224]
	*Commelina*	+fertile	+	plicate		[225]
	*Dichorisandra*	+fertile	+	plicate		[226]
	*Gibasis*	+fertile	+	plicate		[227]
	*Plowmanianthus*	+fertile	+	plicate		[228]
	*Tinantia*	+fertile	+	plicate		[229]
	*Tradescantia*	+fertile	+	plicate		[223]
Pontederiaceae (2)	*Pontederia*	+(fertile in some species)	+fertile	plicate		[60,230,231]
Haemodoraceae (15)	*Lachnanthes*	+	+fertile	ascidiate		[232]
	*Wachendorfia*	+	+fertile	ascidiate *		[232]
**ZINGIBERALES** No data on: Strelitziaceae						
Lowiaceae (1)	*Orchidantha*	+fertile	+	plicate		[233,234]
Heliconiaceae (1)	*Heliconia*	+	+fertile	plicate		[235]
Musaceae (3)	*Musa*	+fertile	+	plicate		[236]
Cannaceae (1)	*Canna*	+fertile	+	plicate		[237,238]
Marantaceae (29)	*Calathea*	+	+fertile	plicate		[237]
	*Ischnosiphon*	+	+fertile	plicate		[237]
	*Thalia*	+	+fertile	plicate		[239]
Costaceae (8)	*Costus*	+fertile	+	plicate		[240]
Zingiberaceae (57)	*Alpinia*	+fertile	+	plicate		[241]
	*Curcuma*	+fertile	+	plicate		[242]
	*Gagnepainia*	+fertile	+	plicate		[243]
	*Globba*	+fertile	+	plicate		[243,244,245]
	*Hedychium*	+fertile	+	plicate		[246,247]
	*Hemiorchis*	+fertile	+	plicate		[243]
	*Scaphochlamys*	+fertile	+	plicate		[248]
	*Zingiber*	+fertile	+	plicate		[249]
**POALES** No data on: Flagellariaceae						
Typhaceae (2)	*Sparganium*	+	+fertile	ascidiate		[72,91]
	*Typha*	+	+fertile	ascidiate		[250,251,252]
Bromeliaceae (76)	*Aechmea*	+fertile	+fertile	plicate		[253,254]
	*Dyckia*	+fertile	+fertile	plicate		[253]
Rapateaceae (17)	*Duckea*	+	+fertile	plicate		[255]
	*Guacamaya*	+ fertile	+	plicate		[255]
	*Monotrema*	+	+fertile	plicate		[255]
	*Potarophytum*	+	+fertile	plicate		[255]
	*Saxofridericia*	+fertile	+	plicate		[255,256]
	*Spathanthus*	+	+fertile	plicate		[255]
Xyridaceae (5)	*Abolboda*	+fertile	+fertile	ascidiate		[75]
	*Orectanthe*	+fertile	+	plicate		[75]
	*Xyris*	+fertile	+fertile	plicate/ascidiate		[63,64,257]
Eriocaulaceae (7)	*Eriocaulon*	+	+fertile	ascidiate		[257,258,259]
	*Leiothrix*	+	+fertile	ascidiate		[260]
	*Paepalanthus*	+	+fertile	ascidiate		[261]
Mayacaceae (1)	*Mayaca*	+fertile	−	plicate		[262]
Thurniaceae (2)	*Prionium*	+fertile	+fertile	ascidiate *		[263]
	*Thurnia*	+	+fertile	ascidiate *		[264]
Juncaceae (8)	*Juncus*	+fertile	+fertile	ascidiate *		This study
Cyperaceae (94)!	*Abildgaardia*	+	+fertile	ascidiate *		[265]
	*Bulbostylis*	+	+fertile	ascidiate *		[265,266]
	*Cyperus*	+	+fertile	ascidiate *		[72,91]
	*Dulichium*	+	+fertile	ascidiate *		[267,268]
	*Eleocharis*	+	+fertile	ascidiate *		[269]
	*Eriophorum*	+	+fertile	ascidiate *		[267]
	*Exocarya*	+	+fertile	ascidiate *		[270]
	*Ficinia*	+	+fertile	ascidiate *		[271]
	*Fimbristylis*	+	+fertile	ascidiate *		[265]
	*Fuirena*	+	+fertile	ascidiate *		[272]
	*Hellmuthia*	+	+fertile	ascidiate *		[273]
	*Hypolytrum*	+	+fertile	ascidiate *		[274]
	*Isolepis*	+	+fertile	ascidiate *		[271]
	*Lepironia*	+	+fertile	ascidiate *		[275]
	*Mapania*	+	+fertile	ascidiate *		[274]
	*Pycreus*	+	+fertile	ascidiate *		[268]
	*Rhynchospora*	+	+fertile	ascidiate *		[276]
	*Scirpus*	+	+fertile	ascidiate *		[72,91,267,268]
Restionaceae s.str (42)	*Cannomois*	+	+fertile	ascidiate		[277]
	*Ceratocaryum*	+	+fertile	ascidiate		[277]
	*Chondropetalum*	+	+fertile	ascidiate		[278]
	*Dovea*	+	+fertile	ascidiate		[278]
	*Elegia*	+	+fertile	ascidiate		[278]
	*Hydrophilus*	+	+fertile	ascidiate		[277]
	*Hypodiscus*	+	+fertile	ascidiate		[277]
	*Ischyrolepis*	+	+fertile	ascidiate		[278]
	*Nevillea*	+	+fertile	ascidiate		[277]
	*Restio*	+	+fertile	ascidiate		[278]
	*Staberoha*	+	+fertile	ascidiate		[278]
	*Thamnochortus*	+	+fertile	ascidiate		[278]
Restionaceae-Anarthriaceae (3)	*Anarthria*	+	+fertile	ascidiate		[46]
	*Hopkinsia*	+	+fertile	ascidiate *		[46]
	*Lyginia*	+	+fertile	ascidiate		[46]
Restionaceae-Centrolepidaceae (3)	*Aphelia*	+	+fertile	ascidiate *		[279]
	*Centrolepis*	+	+fertile	ascidiate *		[279,280]
Joinvilleaceae (1)	*Joinvillea*	+	+fertile	ascidiate *		[281]
Ecdeiocoleaceae (2)	*Ecdeiocolea*	+	+fertile	?		[282]
Poaceae (793)!		+	+fertile	ascidiate? *		[43] and references therein

## 3. Materials and Methods

An extensive search of published data on monocot flower development was performed. The data on carpel structure and development are presented in Table 1. We mainly focused on publications that used scanning electron microscopy (SEM), because this method allows for better documentation of the results.

For novel observations, plant material was fixed and stored in 70% ethanol. For SEM, flowers at different developmental stages were dissected in 96% ethanol under an Olympus SZX7 stereomicroscope (Tokyo, Japan), dehydrated through absolute acetone, critical-point dried using a Hitachi HCP-2 critical-point drier (Tokyo, Japan), then coated with gold and palladium using an Eiko IB-3 ion-coater (Tokyo, Japan). Observations were made using a CAMSCAN S2 SEM (Camscan, Cambridge, UK) at the Laboratory of Electron Microscopy of the Biological Faculty of Moscow State University.

Parsimony reconstructions of the evolution of carpel characters were preformed using WinClada version 1.00.08 [28]. The character of carpel development was binary, but the two characters of carpel structure were multistate (with three states each). The character of the presence of carpel zones was analysed as ordered, implying that transitions between completely ascidiate and completely plicate carpels should occur via the condition with both carpel zones present. The character of carpel zone fertility was analysed as unordered, because there are clades where carpels possess single ovules, for which a condition with both carpel zones fertile is not allowed. It should be stressed, however, that the interpretation of both characters as ordered vs. unordered resulted in only minor differences in parsimony reconstructions (namely, it was ambiguous with carpel zone is fertile in the common ancestor of Haemodoraceae and Pontederiaceae when the character was analysed as ordered; the common ancestor of Aponogetonaceae and its sister clade was inferred as having only (sym)plicate zone when the character was analysed as unordered).

The material was first assembled at the genus level (Table 1). Polymorphisms were used in character coding in the few cases when differences among various species of a genus are documented. Gynoecia of some Araceae and *Ruscus* (Asparagaceae) can be interpreted as either monomerous or pseudomonomerous. Both interpretations are provided in Table 1, but the monomerous one is used for the analysis of character evolution, because the evidence of rudiments of the regular occurrence of reduced carpels is not completely convincing.

We used the plastid phylogenomic analysis of Givnish et al. [29] as the main source of the plastid tree topology used here. Terminal groups were adjusted to our data set on carpel structure and development. When all members of a family are uniform in all our characters of interest (at the current state of knowledge), the family was used as a terminal group. In a few families with non-uniform carpel structure and development, taxon sampling of Givnish et al. [29] was insufficient to cover all genera of our data set. The following sources, all based on plastid markers or plastomes were used for such families Araceae [30], Amaryllidaceae [31], Asparagaceae [32,33], Arecaceae [34], Rapateaceae [35]. *Duckea* (Rapateaceae) was excluded from the analysis of character evolution, because we found no plastid data on its phylogenetic placement. Relationships within the clade of Burmanniaceae and Dioscoreaceae are shown as unresolved, because no phylogenomic data are available for *Stenomeris* and the data on the placement of *Gymnosyphon* are controversial [36,37]. Relationships among the three genera of Xyridaceae sampled here are set as *Xyris* (*Abolboda* + *Orectanthe*). Michelangeli et al. [38] showed that *Abolboda* is sister to *Orectanthe*, though *Xyris* was not sister to this clade in their analysis. Givnish et al. [29] supported monophyly of Xyridaceae, though they studied *Abolboda* and *Xyris*, but not *Orectanthe*.

The most representative nuclear phylogenomic analysis of monocots includes 192 species and 72 of 77 monocot families [39]. It has some genus-level sampling gaps relevant to the present study. We constructed a tree topology where all relationships of the orders and families were set according to nuclear phylogenomic data [38], but relationships within the families are as in the plastid tree. Three families (Blandfordiaceae, Corsiaceae, Ruppiaceae) were excluded from the tree, because they were not sampled in the nuclear phylogenomic analysis [38]. The nuclear and plastid phylogenies of monocots are largely congruent. The main features of the nuclear tree relevant to the present study are the position of Tofieldiaceae as sister to the rest of Alismatales and the position of Typhaceae as sister to the rest of Poales.

## 4. Conclusions and Outlook

Our data strongly suggest that the common ancestor of monocots had carpels with both zones (ascidiate and plicate) and late peltation in ontogeny (the plicate zone was first to initiate). This result was found irrespective of the use of the plastid (Figure 2 and Figure 4) or nuclear (Figure 3 and Figure 5) phylogeny. What carpel zone(s) was ancestrally fertile in monocots remains unclear, partly because of the unusual gynoecium morphology of the sister to all other extant monocots, *Acorus* [39,61]. At least under family inter-relationships found using nuclear data, the clade sister to *Acorus* is characterised by the ancestral gynoecium condition with only (sym)plicate zone fertile (Figure 3).

Further progress in understanding patterns of carpel evolution could be achieved by the use of more sophisticated approaches such as maximum likelihood and Bayesian analysis using time-calibrated trees. We believe, however, that it is equally or even more important to fill the considerable gaps remaining in the primary knowledge of gynoecium structure and development across monocots. Apparently, the sampling of developmental studies has been biased by attention to taxa with unusual flower construction. For example, the species-poor but morphologically diverse group of core Alismatales is quite well represented in the present data set. In contrast, developmental data on the order Liliales are clearly insufficient. We found no published data on flower development in six of the ten currently recognised families of the order. There is a tendency that the model-based methods leave some important questions of morphological evolution unresolved, especially when groups near the base of a phylogeny are morphologically heterogenous. For example, even with the use of model-based methods, it is unclear whether the ancestral angiosperm gynoecium has free (apocarpy) or fused (syncarpy) carpels (see [19,90]). We advocate that the use of multiple lines of evidence, including comparative and developmental morphology, developmental genetics, phylogeny-based approaches and palaeobotany will resolve the picture of the origin and subsequent evolution of the angiosperm gynoecium.

## Data Availability

The original contributions presented in the study are included in the article.
